# Privacy-preserving data quality assessment for federated health data networks

**DOI:** 10.1186/s12911-025-03328-6

**Published:** 2026-01-27

**Authors:** Radovan Tomášik, Tobias Kussel, Zdenka Dudová, Radoslava Kacová, Roman Hrstka, Martin Lablans, Petr Holub

**Affiliations:** 1 Faculty of Informatics, Masaryk University, Botanicka, 68a, Brno, Jihomoravsky Kraj, 60200 Czechia; 2 Bank of Biological Material, Masaryk Memorial Cancer Institute, Zluty Kopec 7, Brno, Jihomoravsky Kraj 60200 Czechia; 3 Federated Information Systems, German Cancer Research Center (DKFZ), Im Neuenheimer Feld 280, 69120 Heidelberg, Germany; 4 Complex Medical Informatics, Medical Faculty Mannheim, Heidelberg University, Theodor-Kutzner-Ufer 1–3, 68167 Mannheim, Germany; 5 BBMRI-ERIC, Neue Stiftingtalstrasse, 2/B/6, Graz, 8010 Austria; 6 Institute of Computer Science Masaryk University, Botanicka 68a, Brno, Jihomoravsky Kraj 60200 Czechia

**Keywords:** Differential privacy, Data quality, Federated data, Medical informatics, BBMRI, CQL

## Abstract

**Background:**

Assessing data quality in federated health data systems presents unique challenges, particularly when data custodians cannot expose raw data due to privacy regulations. Traditional quality assessment approaches often require centralised access, which conflicts with the principles of data sovereignty and confidentiality.

**Methods:**

In this study, we evaluate the utility of federated data quality assessment with differential privacy techniques to safeguard sensitive health data. The aim is to develop tooling and demonstrate a proof-of-concept implementation over a synthetic dataset of observational medical data.

**Results:**

We present a privacy-preserving framework for evaluating data quality in federated environments using differential privacy. Our approach enables individual data providers to compute local quality metrics and share only aggregated, privacy-protected results. We implement a proof-of-concept that supports predefined quality checks across different data models and demonstrate how meaningful insights into data quality can be obtained without compromising sensitive information.

**Conclusion:**

This work demonstrates that differential privacy can be effectively applied to enable federated quality assessment in health data networks without compromising individual privacy. By implementing a proof-of-concept system over synthetic health data, we show that it is possible to obtain meaningful quality metrics in a decentralised setting.

## Background

In recent years, biomedical research has become increasingly data-driven, placing new demands on the institutions that generate and manage research-relevant data, such as hospitals, biobanks, and other biomolecular repositories [1]. This evolution has been accompanied by the growth of large-scale studies that often require the integration of data from multiple, heterogeneous sources—a process that is both technically complex and time-consuming [2]. Traditionally, this integration follows a centralised model, where data from different providers is aggregated into a single repository. However, a complementary approach has been gaining traction: decentralised or federated data repositories, in which data remains within its original institution but can be queried or analysed across nodes. Each of these models offers distinct benefits and trade-offs in terms of scalability, control, and privacy. Regardless of the architecture, however, both centralised and decentralised approaches must confront the fundamental challenge of ensuring adequate data quality across diverse and distributed datasets for processing purposes [3].

The concept of data quality—or fitness for purpose^1^—is as essential in federated systems as it is in centralised ones [4]. Although the underlying infrastructures differ, the key dimensions used to evaluate data quality remain consistent across both paradigms. These dimensions offer a structured framework for determining whether a dataset is suitable for specific analytical tasks: a consideration of particular importance in federated environments where data remains distributed and the data user may only be allowed to send the algorithm to the data without ever seeing the data. Table 1 summarises the most commonly referenced data quality dimensions, along with brief descriptions. These were identified through a review of key literature on data quality frameworks and assessment methodologies [5–8].

**Table 1:** Data quality dimensions with brief descriptions

**Dimension**	**Description**
Accuracy	The degree to which data correctly reflects the real-world objects or events it represents.
Completeness	The extent to which all required data is available and captured.
Consistency	Ensures that data is uniform across systems and does not conflict within itself.
Timeliness	The extent to which data is up-to-date and available within a useful timeframe.
Validity	Ensures data conforms to the required format, standards, or constraints.
Uniqueness	Ensures that there are no unnecessary duplicates in the data.
Reliability	The degree to which data is dependable and trustworthy for decision-making.
Relevance	The extent to which data is applicable and useful for the specific purpose or context.
Accessibility	Ensures data is available and retrievable when needed, given appropriate authorisation.
Interpretability	The degree to which data is clear and easy to understand, with sufficient metadata or context provided.

Access to health data for secondary use remains challenging due to its sensitive nature and the presence of legal and regulatory barriers designed to prevent misuse [9]. Yet, secondary use of health data is critical for advancing research, improving healthcare delivery, and informing public health policy [10]. In response, the FAIR principles—ensuring that data are Findable, Accessible, Interoperable, and Reusable—have gained significant traction as a framework to promote data sharing and reuse [11]. However, while these principles address key aspects of data governance, they do not inherently guarantee the quality of the underlying data. A dataset that is incomplete, inaccurate, or contains duplicates may fully comply with FAIR principles yet remain unsuitable for meaningful analysis as FAIR principles do not inherently ensure high data quality [12]. This underscores a critical gap addressed by the FAIR-Health principles [13]: the need for systematic data quality documentation and assessment. This study aims to investigate how such data made discoverable through a federated querying system—such as the BBMRI-ERIC Locator [14]—can utilise federated data quality checks in a privacy-preserving manner. Ideally, the results of such quality assessment could be disclosed semi-publicly. By semi-publicly, we refer to users of the federated querying system that have been identified with a certain level of trust and have agreed to adequate acceptable use policy of the system. This is a typical requirement for processing anonymised data bearing small but non-zero residual privacy risk. Our approach enables an overview of data quality at individual nodes, without requiring the central node to have access to the raw data itself. Privacy preservation is crucial in that semi-public context because even aggregated quality metrics can inadvertently expose sensitive information. For example, a patient with a rare diagnosis or an uncommon combination of attributes could be reidentified if detailed quality checks reveal small counts or unique patterns.

Data quality assurance metrics are a requirement for maintaining high-quality data, hence many generic solutions exist. The HL7 Clinical Quality Language (CQL) is a standard allowing the definition of complex quality measures and analyses on data adhering to the HL7 FHIR data model and standard [15]. Providing a complete query language enables sophisticated and purpose-built quality checks, however, being restricted to FHIR-based data and not providing a default set of usable quality checks severely limits portability across projects and networks. Another relevant example of large-scale, automated data quality assessment is the OHDSI Data Quality Dashboard (DQD), in contrast to HL7 CQL a graphical application, which evaluates datasets structured under the OMOP Common Data Model using more than 3000 predefined checks spanning conformance, completeness, and plausibility [16]. The DQD is widely adopted across international research networks and is typically executed locally by each data custodian, allowing sites to assess and document data quality without exposing patient-level information. Because the DQD is tightly coupled to OMOP and relies on SQL-based rule execution, it is not readily adaptable to other data structures or modalities—such as imaging, genomic profiles, or custom data models that require validation via Python scripts or alternative processing pipelines. Lastly, within BBMRI-ERIC a site-local data quality reporting system for biosample data is being developed, providing data custodians with technical and statistical quality reports [17]. Although these decentralised execution align with the principles of data sovereignty in federated environments, the presented solutions do not incorporate mechanisms for privacy-preserving disclosure of results. Sites must manually decide which aggregated metrics can be shared, and summary values may still risk unintended disclosure for small cohorts or rare conditions. These characteristics underscore the need for a complementary approach, such as the one developed in this study, that supports federated execution, formal differential privacy guarantees, and extensibility across heterogeneous data types.

### Differential privacy

One widely adopted approach to safeguarding privacy in statistical analysis and disclosure is *differential privacy* [18], which offers strong mathematical guarantees against the reidentification of individuals on the level of algorithm through which the data is “observed”, e.g., by introducing controlled perturbations (e.g. noise) into query results or model parameters [19]. While differential privacy is highly effective for protecting sensitive information, like any anonymisation technique, it introduces trade-offs that can hinder the utility of the analysis. The noise injected to preserve privacy can distort key statistical properties of the data, making it difficult to accurately evaluate completeness, consistency, or accuracy, especially in small or sparse datasets [18].

Formally, a randomised mechanism $\mathcal{M}$ is said to satisfy $(\varepsilon, \delta)$-*differential privacy* if the following condition holds:

1 $$\Pr[\mathcal{M}(D_1) \in S] \leq e^{\varepsilon} \cdot \Pr[\mathcal{M}(D_2) \in S] + \delta$$

for all datasets $D_1$ and $D_2$ with an $\ell_1$ distance $||x-y||_1\leq 1 \ (x\in D_1, y\in D_2)$, and for all measurable subsets $S$ of the output space of $\mathcal{M}$. For example, for tabular datasets, the distance $\ell_1 \leq 1$ means that they differ in at most one record.

Here, $\varepsilon$ known as “privacy budget”, quantifies the privacy loss, with smaller values indicating stronger privacy, while $\delta$ allows for a small probability of the guarantee being violated. This definition ensures that the presence or absence of any single individual in the dataset, regardless of how unique this individual is, has a limited and quantifiable effect on the output of the mechanism.

As a simple example of a data quality check, let $f(D)$ represent the number of Patient entities with a recorded date of birth. To preserve privacy, the reported result is:

$$f^{\prime}(D) = f(D) + \mathrm{Lap}\left(\frac{\Delta f}{\varepsilon}\right)$$

where $\mathrm{Lap}\left(\frac{\Delta f}{\varepsilon}\right)$ denotes noise drawn from the Laplace distribution, calibrated to the sensitivity $\Delta f$ of the query and the chosen privacy parameter $\varepsilon$. For simple queries, counting the number of records (i.e., patients) $\Delta f = 1$.

A single application of a differentially private mechanism to a quality check (e.g., verifying the presence of birth dates in Patient records) ensures privacy with respect to that individual computation. However, in realistic scenarios involving complex data models, multiple such analyses are often required. Applying differential privacy independently to each quality check would lead to a cumulative privacy loss, which must be properly accounted for.

This cumulative effect is governed by the *sequential composition theorem* [20]. Specifically, if $k$ mechanisms $\mathcal{M}_1, \dots, \mathcal{M}_k$ are applied sequentially to the same dataset $D$, where each mechanism $\mathcal{M}_i$ satisfies $\varepsilon_i$-differential privacy, then the sequence of all $k$ computations satisfies:

$$\left(\sum_{i=1}^k \varepsilon_i\right)\text{-differential privacy.}$$

In practice, this means that the total privacy budget must be carefully distributed across all quality checks to maintain an acceptable overall privacy guarantee. When further granularity is required—such as stratification by categories (e.g., age groups, gender, condition types)—the number of queries increases, leading to additional privacy cost due to the sequential composition property of differential privacy.

Let $f(D)$ be a query function over dataset $D$, and let the mechanism $M(D)$ be defined using the Laplace mechanism as:

$$M(D) = f(D) + \mathrm{Lap}\left(\frac{\Delta f}{\varepsilon}\right)$$

Introducing stratification results in multiple such queries $f_1(D), f_2(D), \dots, f_k(D)$, each corresponding to a stratum. According to the sequential composition theorem, the total privacy budget used becomes:

$$\varepsilon_{\mathrm{total}} = \sum_{i=1}^k \varepsilon_i$$

For uniform distribution of the privacy budget across the composition to preserve the fixed total privacy budget $\varepsilon_{\mathrm{total}}$, each stratum must use:

$$\varepsilon_i = \frac{\varepsilon_{\mathrm{total}}}{k}$$

Consequently, the Laplace noise added to each result is scaled as:

$$\mathrm{Lap}\left(\frac{\Delta f \cdot k}{\varepsilon_{\mathrm{total}}}\right)$$

This leads to a quadratic increase in variance with respect to the number of strata:

$$\mathrm{Var}\left[\mathrm{Lap}\left(\frac{\Delta f \cdot k}{\varepsilon_{\mathrm{total}}}\right)\right] = 2 \left(\frac{\Delta f \cdot k}{\varepsilon_{\mathrm{total}}}\right)^2$$

Thus, stratifying quality checks enables more granular and informative insights by ensuring balanced representation across key subgroups, which improves the precision and interpretability of results [21]. However, this increased granularity comes at the cost of higher variance in individual measurements due to the privacy budget being divided among a larger number of stratified queries^2^. This trade-off reduces the utility of each measurement and must be carefully considered when designing privacy-preserving data quality frameworks. In addition to this general limitation, one must also consider how low counts within specific strata are affected. Stratification can result in very small subgroups, which are impacted by differential privacy in much the same way as small datasets overall. For instance, conditions like cancer may be rare in younger populations, while the number of individuals over 90 years old is typically limited. In both cases, the small subgroup size amplifies the impact of the injected noise, reducing the interpretability of the results. A more detailed discussion of these limitations, along with potential mitigation strategies, is provided in the *Discussion* section.

Conversely, reducing the number of strata decreases the noise added per result, highlighting the need to balance the level of stratification with the desired accuracy.

### Research objective

Our aim is to leverage differential privacy as a mechanism for assessing and communicating the quality of underlying datasets in a privacy-preserving manner, thereby enabling effective quality assessment without requiring direct access to the raw data.

The paper begins by introducing the problem domain, followed by a detailed description of the methods employed in the study. It then proposes and implements a conceptual framework for assessing the quality of health data in a federated environment, incorporating differential privacy techniques to safeguard the resulting metrics.

## Methods

This study focused on implementing data quality checks for the first six dimensions listed in Table 1. These dimensions were selected due to their expressiveness and suitability for objective, automated evaluation. In contrast, the remaining dimensions—Relevance, Accessibility, and Interpretability—were excluded as they are inherently subjective and challenging to quantify programmatically. The proposed quality checks were initially validated on a synthetic dataset designed to emulate real-world pseudonymised health data, structured according to the HL7 FHIR (Fast Healthcare Interoperability Resources) standard [22]. The dataset comprises 1,000 Patient resources and 10,000 Specimen resources and is meant to depict a general cohort of patients without any specific focus or grouping.

The proposed framework currently relies on a predefined set of data quality checks. These were selected to reflect real-world data issues based on the authors’ practical experience with the BBMRI-ERIC Federated Search platform^3^. The nine checks used in this study (listed in Table 2) were chosen to demonstrate coverage of key dimensions such as accuracy and completeness while remaining general enough to be applicable across multiple datasets.

**Table 2:** Data quality checks for FHIR resources with a description, dimension, raw value and a value obfuscated with differential privacy

Check ID	Description	Dimension	Raw Value [%]	DP Value [%]
accuracy-1	How many patients have an incompatible Diagnosis (e.g. Prostate Cancer for Female)$^{3}$	Accuracy	0.10	0.70
accuracy-2	Date of Birth before 1900 or in the future	Accuracy	5.60	5.60
completness-1	How many patients have missing gender info	Completeness	3.70	4.90
completness-2	How many patients do not have a condition	Completeness	20.00	20.10
consistency-1	How many patients do not have a supported gender value	Consistency	9.60	10.80
timeliness-1	How many patients were last updated more than a year ago	Timeliness	0.00	0.00
validity-1	How many patients have conditions with invalid ICD-10 codes	Validity	96.90	96.80
uniqness-1	How many patients are duplicated in the dataset based on IDs	Uniqueness	9.70	9.60
accuracy-3	Survival rate for female patients	Accuracy	27.60	26.30
accuracy-3	Survival rate for male patients	Accuracy	30.90	32.00

Each check was first defined at a conceptual level, for example, detecting duplicate patient IDs, and then implemented using either Clinical Quality Language (CQL) or Java functions leveraging the FHIR API. The current implementation supports dynamic editing and addition of new CQL-based checks within the data quality agent, provided that the target FHIR store supports CQL execution. Therefore, the framework is not limited to these nine checks; rather, they serve as a representative subset for this proof-of-concept. Future work will focus on expanding the check library and supporting more complex or domain-specific validations.

To ensure differential privacy, noise drawn from a Laplace distribution was added to the resulting values of each check. Both raw and obfuscated results were stored locally, but only the privacy-preserving values were exposed externally. Retaining the unmodified results enables internal monitoring of privacy impact and supports local troubleshooting. To evaluate the effectiveness of the privacy-preserving approach, the obfuscated results obtained from the synthetic dataset were compared against baseline values calculated without the application of differential privacy. The synthetic dataset conforms to the FHIR-based data model, which is publicly available via Simplifier.net [23]. The data was maintained in a FHIR-compliant data repository [24], and quality assessments were performed using a combination of Clinical Quality Language (CQL) queries and Java-based programmatic functions leveraging the FHIR API. This approach facilitated standardised and platform-independent querying across different FHIR stores.

The utility of each data quality check under differential privacy was assessed by comparing its output to the corresponding raw (non-privatised) value. In this proof-of-concept implementation, each individual check was allocated a privacy budget of $\varepsilon = 0.2$, while the stratified check received a slightly higher budget of $\varepsilon = 0.3$ due to its increased utility demands. The total privacy budget for the entire report was capped at $\varepsilon = 2.0$, ensuring that the cumulative privacy loss across all checks did not exceed this limit. The framework is designed to support flexible budget allocation depending on the context; the specific values used here were selected to demonstrate the feasibility of the approach within a constrained privacy budget.

All individual quality checks in this study were designed to evaluate data at the patient level. Accordingly, the sensitivity parameter $\Delta f$, which determines the maximum change in a query’s output resulting from the addition or removal of a single individual, was set to 1. This reflects the assumption that each patient contributes to the metric at most once. However, in scenarios where quality checks target data at a finer granularity, such as individual samples rather than patients, $\Delta f$ must be adjusted to account for the potential contribution of multiple records per individual. In such cases, to maintain meaningful privacy guarantees, $\Delta f$ should be set to the maximum number of samples that any single patient contributes within the dataset. This ensures that the differential privacy mechanism still prevents inferences about any particular patient, even when multiple data points per individual are involved.

## Results

This study designed and practically implemented a conceptual framework for implementing data quality checks within federated data systems in a privacy-preserving manner. In this framework, quality metrics are computed locally at each federated node and subsequently made visible in a central analysis interface. While this enables aggregate insights across the network, it also introduces privacy risks. For example, low or rare metric values, such as a single patient with an unusual diagnosis–gender mismatch^4^, could potentially lead to reidentification or linkage attacks. To mitigate these risks, the framework applies differential privacy to each local report prior to its publication. By calibrating noise to the sensitivity of each metric and enforcing a per-node privacy budget, the approach protects individual-level data while still enabling meaningful, aggregated quality assessments.

The first step in validating this framework involved designing specific data quality checks corresponding to the first six dimensions listed in Table 1, tailored to the structure and semantics of the synthetic dataset’s data model. Table 2 summarises the types of checks implemented for each dimension. The complete implementation, which utilises a range of tools to perform the defined quality assessments, is available on GitHub [25]. The system was developed using Java and the Spring framework for backend functionality, and a Vue.js-based interface for the frontend. Interaction with the underlying FHIR store is facilitated through the HAPI FHIR library, complemented by custom HTTP requests to support more complex operations.

**Fig. 1:**
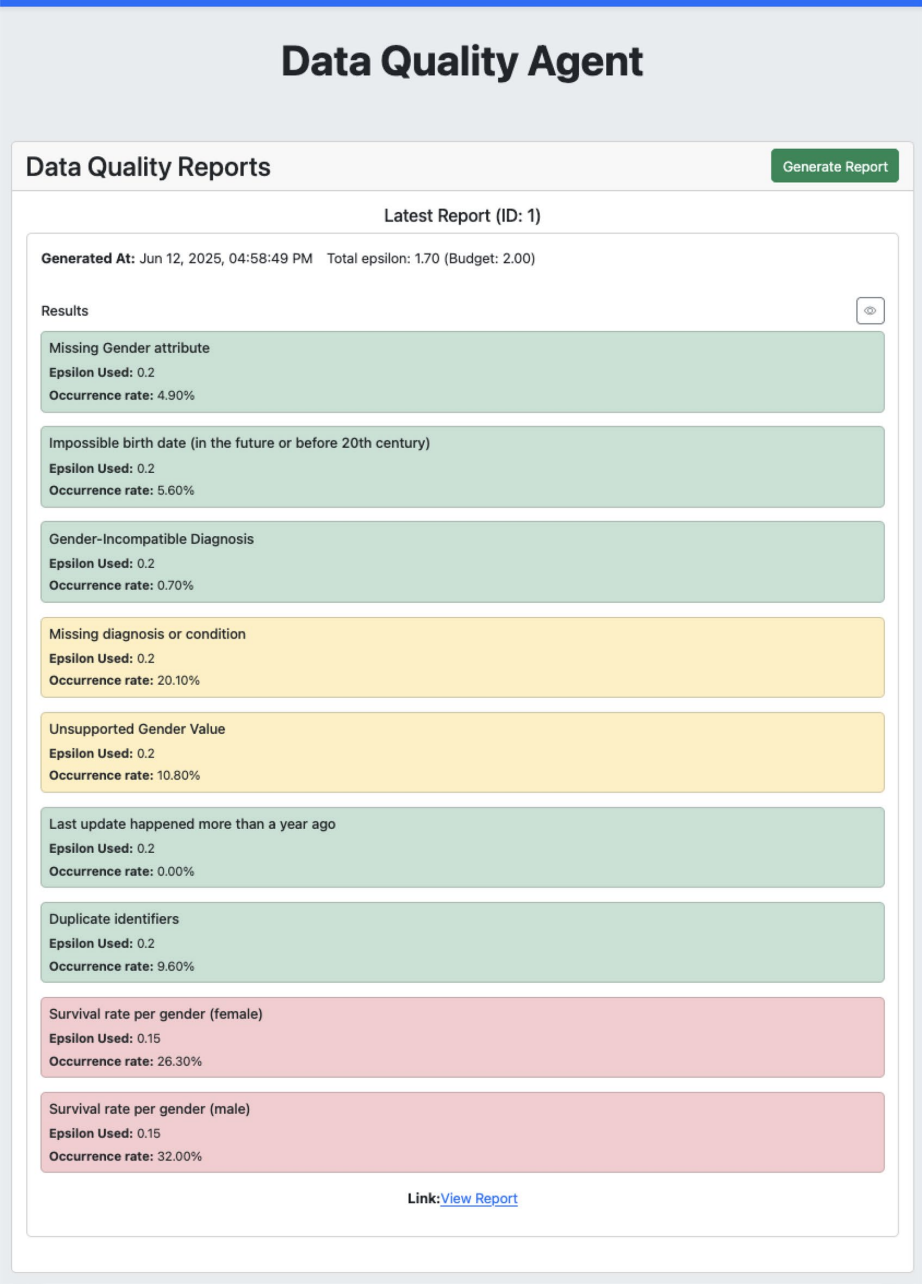
Data quality check report. For a description of the privacy budget, please refer to the paragraphs above

The created quality checks were executed against the synthetic dataset available on GitHub; the synthetic data was generated using the bbmri-fhir-gen [26] tool, which purposefully injects data quality errors. Table 2 shows the resulting quality check report from a single run, summarising the results of the quality checks with raw results and results with applied differential privacy^5^. As visible in Fig. 1, the total privacy budget was set to $\varepsilon = 2.00$. In this example, the allocation across checks was manually adjusted to demonstrate the dynamic configuration capabilities of the DQMA. Specifically, each check was assigned an individual $\varepsilon$ value such that the total used budget summed to $\varepsilon = 1.70$. For instance, one check received $\varepsilon = 0.30$ to support stratified reporting (accuracy-3 survivable rate by gender)^6^, while others were allocated lower budgets of $\varepsilon = 0.20$. This example intentionally deviates from uniform allocation to highlight how privacy budgets can be tuned per check based on sensitivity, importance, or user preference—providing flexibility for real-world deployments.

A small difference between the raw and DP value is generally considered acceptable, as it indicates that the noise introduced for privacy preservation does not significantly distort the analytical outcome. Check *accuracy-1* had a raw value of 0.10% with an obfuscated value being 0.70%. With such low numbers, the relative deviation may seem very large (7x increase); however, on a dataset this size, it can still be interpreted as “Less than 1% of patients have an incompatible diagnosis”.

For example, the *completeness-1* check quantifies the number of patients in the dataset missing gender information. In this case, the raw value is 3.70% out of 1,000 patients, and with added noise, this number is 4.90%. To preserve privacy, both the count and the total number of patients may be obfuscated using noise. The reported percentage is then computed as:

$$\text{Obfuscated Percentage} = \left( \frac{\tilde{c}}{\tilde{n}} \right) \times 100$$

where $\tilde{c} = c + \eta_1$ is the obfuscated count, $\tilde{n} = n + \eta_2$ is the obfuscated total number of patients, $c$ is the raw count, $n$ is the true total, and $\eta_1, \eta_2$ are independent noise terms typically drawn from the Laplace distribution. This formulation ensures that both the numerator and denominator incorporate differential privacy protection. The *validity-1* check shows that 96.90% of patients in the dataset have invalid ICD-10 codes. This high proportion is due to the synthetic data generator, which creates ICD-10 codes using a regular expression pattern rather than a controlled terminology.

Currently, there is no mechanism in place to further obfuscate low counts, such as values below 10. However, this can be readily incorporated in any implementation by applying thresholding or rounding such small values down to zero to enhance privacy protection.

As the goal of this endeavour was to generate comprehensive data quality reports, the computed values were then put into a quality check report to increase user friendliness and showcase how such a report can be shared along with metadata of a dataset. Figure 1 shows the UI of the data quality agent with a generated report ready for sharing. Each quality check result is visually encoded using a colour scheme based on configurable threshold values. By default, checks implemented via Clinical Quality Language (CQL) use a warning threshold of 10% and an error threshold of 30%. For example, if more than 10% of records fail a check, the result is shown in yellow (warning); if more than 30% fail, it is shown in red (error). Results below the warning threshold appear in green to indicate acceptable quality. These threshold values were selected for demonstration purposes and can be adjusted individually for each quality check based on project-specific requirements.

**Fig. 2:**
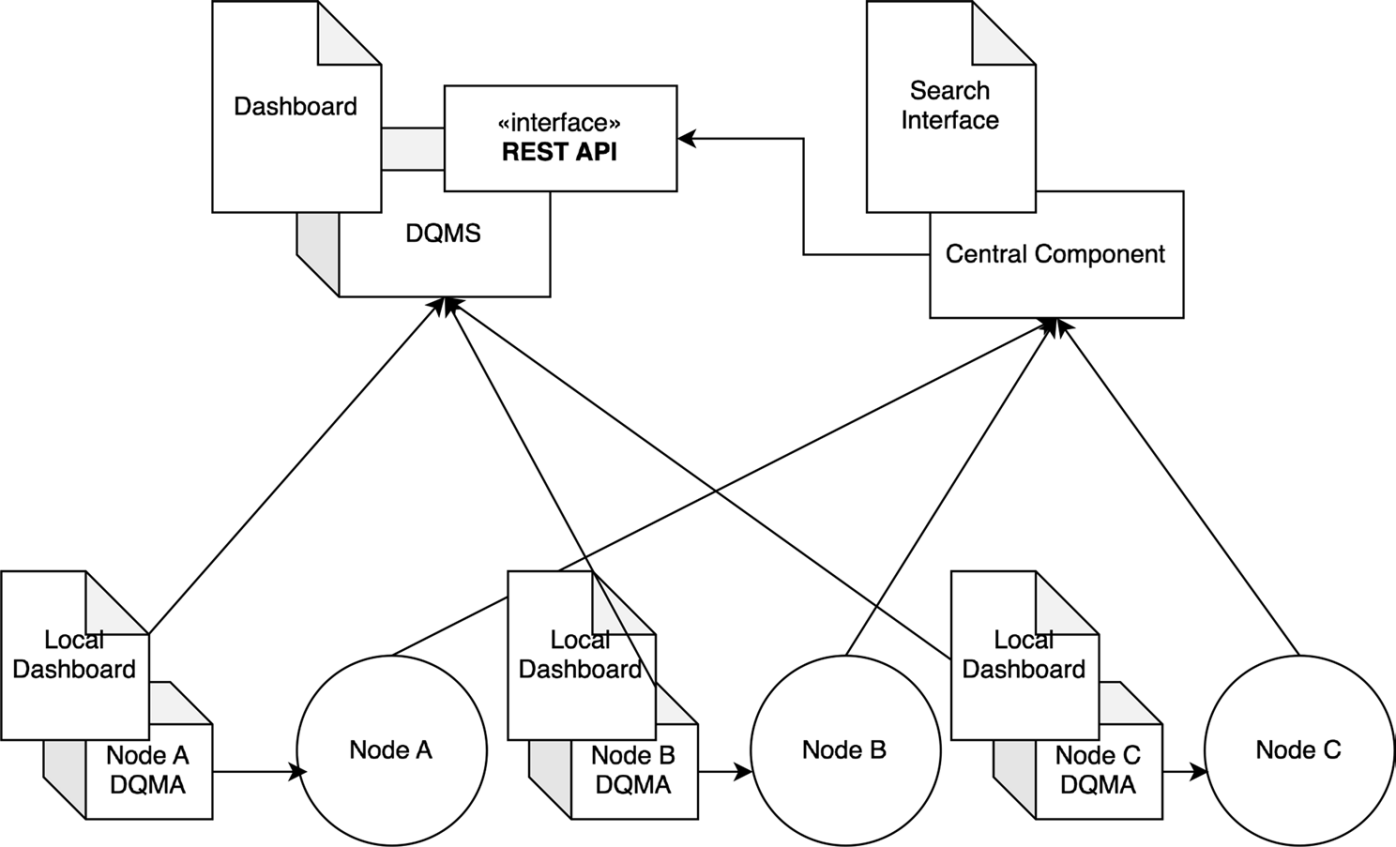
Federated data quality assessment system architecture diagram

To further demonstrate the applicability of the proposed approach, a pilot implementation is currently underway within the federated search system of the European Research Infrastructure for Biobanking and Biomolecular Resources (BBMRI-ERIC) [27]. While tools for authorised personnel to locally generate non-privatised quality reports already exist [28], the quality checks developed in this study, aiming for semi-public disclosure, are being deployed across individual nodes of the federation. Figure 2 presents a high-level overview of the architecture and introduces two key components that enable this functionality: the Data Quality Metrics Agent (DQMA) and the Data Quality Metrics Server (DQMS).

The DQMA operates locally at each node within the federated system. It executes quality checks, collects data quality metrics, and interprets the results locally, ensuring that sensitive information is never exposed outside the local environment. When metrics are transmitted to the DQMS, only numbers obfuscated using differential privacy are sent, such as the report visible in Fig. 1.

The DQMS centrally aggregates and visualises the quality metrics for individual datasets via a configurable dashboard. This server-side component functions independently of the federated system’s core search or analysis interfaces, allowing for easy integration into diverse system architectures. A key aspect of this setup is the definition and configuration of the quality metrics themselves, which can either be maintained locally by the DQMA or retrieved from the DQMS to ensure consistency and manageability across deployments.

## Discussion

The results demonstrate that applying differential privacy to quality checks effectively highlights dataset quality while safeguarding sensitive information. However, the current proof-of-concept has four main limitations.

First, the framework relies on predefined quality checks tailored to a specific data model, which limits flexibility across different health data formats. For example, when applied to OMOP CDM, a dedicated executor must be implemented to translate generic checks (e.g., completeness or conformance) into OMOP-specific SQL queries. While this requires technical adaptation, the core quality concepts remain unchanged. Extending the framework to non-tabular data types, such as medical imaging (e.g., DICOM) or omics data (e.g., VCF), introduces new execution challenges rather than conceptual ones. For instance, image completeness could involve verifying the number of slices or the presence of metadata tags using libraries like *pydicom*, while genomics checks might rely on read-depth thresholds using tools like *vcftools*. The current implementation of the data quality agent supports dynamic editing and execution of CQL queries, providing a degree of flexibility. This points to a clear path forward: defining universal, data-model-agnostic quality checks while implementing format-specific executors to evaluate them. By decoupling quality logic from execution, the framework becomes extensible to structured, unstructured, and multimodal health data sources.

Second, the framework relies on a fixed set of static quality checks, preventing users from creating custom checks on demand. Enabling dynamic, user-defined quality validations would significantly enhance usability but would also require automated evaluation, filtering, and verification mechanisms to maintain consistency and reliability across the system.

Third, by providing a general quality control framework, potential correlations in the individual stratifications can not be exploited, and the total privacy budget calculation defaults to the “conservative” sequential composition. Hence, for specific use cases with known correlations between stratification, the obfuscation might be larger than strictly necessary.

Fourth, while differential privacy provides strong guarantees in large-scale data sharing, its application to small datasets poses significant usability challenges. For example, in a dataset with only 10 patients, even a modest privacy budget (e.g., e = 1) and a sensitivity of 1 can introduce enough Laplace noise to significantly distort results, rendering quality metrics (e.g., completeness or consistency ratios) difficult to interpret. To address this, our framework recommends applying DP only when a minimum cohort size is reached, and suggests aggregating results across sites or time periods to improve robustness. In cases where such aggregation is not possible, alternative privacy-preserving methods or more permissive privacy budgets may be considered, guided by an ethical review of privacy risks versus utility.

Consider computing a data quality metric such as the validity ratio of a particular field (e.g. how many diagnoses have valid ICD-10 codes), defined as $q = \frac{k}{n}$, where $k$ is the number of valid records and $n$ is the dataset size. To ensure $\varepsilon$-differential privacy, Laplace noise is added to $k$, yielding an obfuscated result:

$$\tilde{q} = \frac{k + \mathrm{Lap}(1/\varepsilon)}{n}$$

The standard deviation of the Laplace mechanism is $\mathrm{SD} = \frac{\sqrt{2}}{\varepsilon}$, and the corresponding deviation in the ratio is:

$$\mathrm{SD}[\tilde{q}] = \frac{\sqrt{2}}{n\varepsilon}$$

In small datasets, this deviation becomes significant. For instance, if $n = 10$ and $\varepsilon = 1$, then $\mathrm{SD}[\tilde{q}] \approx 0.141$, meaning that even a result of $q = 0.9$ could be reported as anywhere between 0.35 and 1.0 within a 95% confidence interval. Such noise renders the metric unreliable for downstream use.

Therefore, we recommend applying DP-based noise addition only when $n$ exceeds a minimum threshold (e.g., 30), or aggregating results to increase $n$ without compromising privacy.

One challenge in implementing user-friendly, real-world differential privacy systems is the choice and interpretability of the privacy budget parameter $\epsilon$ [29]. While quantitative frameworks for choosing this parameter exist [30, 31], they are often very complex, involve the estimation of additional (economic) parameters, and don’t foster a more intuitive understanding on the privacy level achieved.

**Fig. 3:**
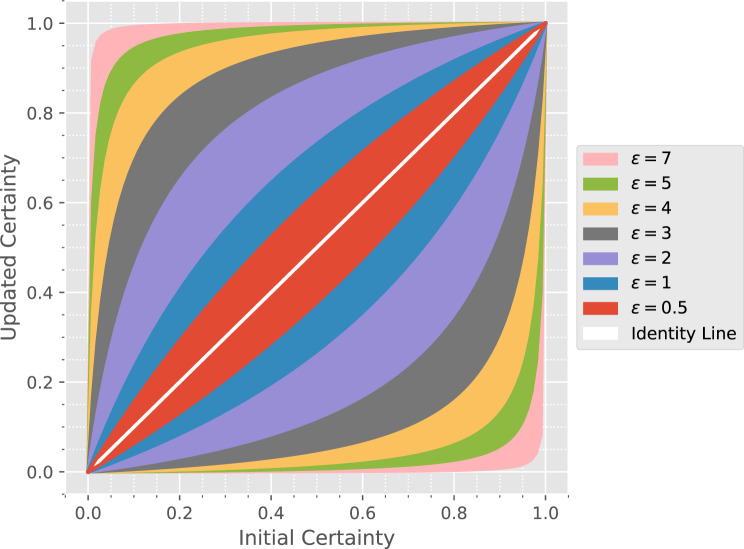
Plot of the initial and updated inclusion certainty for varying privacy budgets $\epsilon$ [32]

To give a more intuitive approach, we follow the argument from Desfontaines and plot the change in certainty that a specific record is included in a data set for varying values of $\epsilon$ [32]. Figure 3 shows the initial certainty of inclusion on the $x$-axis, while the coloured areas indicate the updated certainty bands after one query for specific epsilon values. The sensitivity is set to $\Delta f=1$ for simplicity. The figure demonstrates that even if only a slight initial certainty exists for a record to be part of a data set ($P_\mathrm{in}=0.1$), after even one query with $\epsilon=2$ this could result in an updated certainty of $P_\mathrm{out}\approx 0.45$. This highlights both the need to reduce the number of stratifications and the importance of the ongoing work to develop an optimised differentially private mechanism for specific use cases, exploiting the present correlation.

For a production-ready deployment, the integration of the techniques and components of this work into existing systems, specifically within the BBMRI-ERIC Locator ecosystem, will be pursued as future work [14].

## Conclusion

This proof-of-concept illustrates that differential privacy can be effectively applied to data quality assessments, providing meaningful insights into dataset quality while preserving individual privacy. Although the approach currently requires manual definition of checks per data model and relies on predefined rules, it opens the door to more scalable and flexible solutions. Future work should focus on creating reusable quality checks across common data models and enabling dynamic, user-defined checks supported by automated validation. These developments would significantly enhance the adaptability and usefulness of privacy-preserving quality evaluation frameworks in real-world data sharing scenarios.

## Appendix A Data quality agent screenshots

**Fig. A1:**
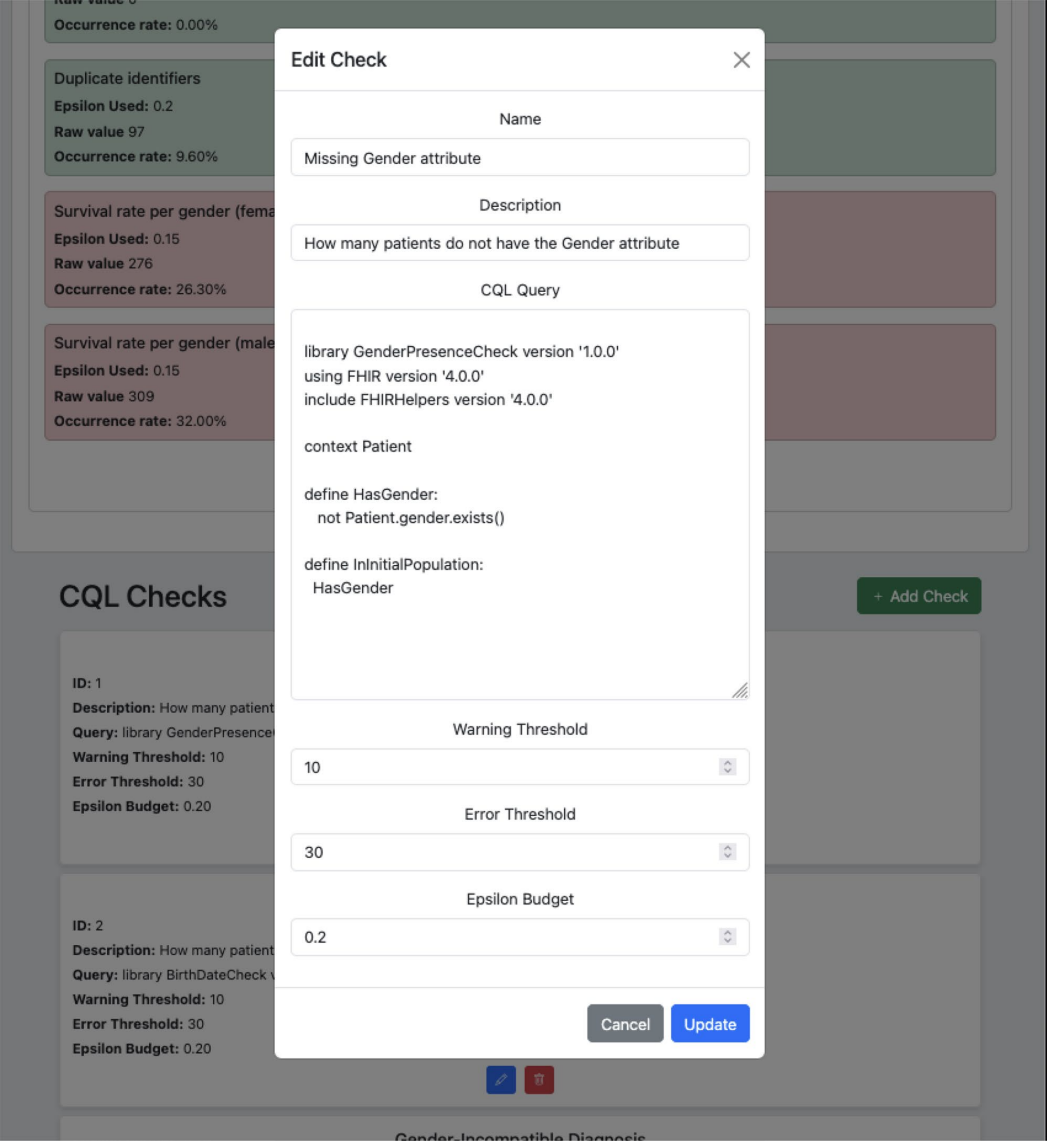
Data quality agent ui for editing CQL checks. User can edit the name, description, query, warning and edit thresholds as well as the epsilon budget allocated to this check

## Data Availability

Synthetic data used in this work is available on GitHub and Zenodo. 10.5281/zenodo.15710278.

## Footnotes

1 Quality is defined as the “degree to which a set of inherent characteristics of an object fulfils requirements” [ISO 9000:2015, 3.6. 2], hence fitness for purpose.

2 Note that the cumulative privacy loss using a Gaussian mechanism is the same under composition.

3 https://locator.bbmri-eric.eu/

4 Note that this refers to *administrative* gender.

5 The DP value is generated by the DQMA, and is just the raw value obfuscated with noise from the Laplace distribution.

6 The privacy budget $\varepsilon$ was divided equally between the two strata (male and female), which is reflected in the figure as the final two quality checks.

## Abbreviations

FAIR: Findable, Accessible, Interoperable, Reusable
FHIR: Fast Healthcare Interoperability Resources
CQL: Clinical Quality Language
BBMRI-ERIC: European Research Infrastructure for Biobanking and Biomolecular Resources
DQMA: Data Quality Metrics Agent
DQMS: Data Quality Metrics Server
